# Using advanced information and communication technologies to advance oncology education in Africa

**DOI:** 10.3332/ecancer.2021.1211

**Published:** 2021-03-23

**Authors:** Lydia Asana, Credit Irabor, Samuel Seppo, Chrystelle Jean, Twalib Ngoma, Ahmed Elzawawy, Wilfred Ngwa

**Affiliations:** 1 Brigham and Women’s Hospital, Dana-Farber Cancer Institute, Harvard Medical School, 75 Francis Street, Boston, MA 02115, USA; 2 University of Central Florida, USA, 4000 Central Florida Blvd, Orlando, FL 32816, USA; 3 Muhimbili University of Health and Allied Science, Tanzania, United Nations Rd, Dar es Salaam, Tanzania; 4 Suez Canal University, Suez Canal Region, Ismailia City, Egypt

**Keywords:** Africa, oncology, e-Learning, information and communication technologies (ICTS)

## Abstract

**Background:**

Recent work has highlighted the tremendous potential of information and communication technologies (ICTs) in advancing global oncology education, research and care. The COVID-19 pandemic has made the development of effective approaches for online education even more crucial. Here we assessed the readiness, interest and potential models for effective implementation of ICT-powered oncology education in Africa.

**Methods:**

Building on previous work by the African Organisation for Research and Training in Cancer (AORTIC), a survey was conducted to assess the electronic learning (e-Learning) readiness of oncology health professionals using an online self-assessment tool. Components of e-Learning readiness assessed include access to computers, Internet, appropriate bandwidth and interest. As a practical test model, an ICT resource-intensive radiation oncology training programme was implemented via the Global Oncology University (GO-U) collaborative education platform. An analysis of results, challenges and opportunities resulting from these is discussed for advancing online oncology education in Africa.

**Results:**

The survey showed over 92% of health professionals have access to computers, laptops or other technology that can allow them to participate in online education. Over 45% of oncology health professionals have already participated in some form of online education. Interest in online education was over 93%. Models for effective online learning in oncology include synchronous and asynchronous short-term courses for continuous education and long-term degree and residency programmes. There was a significant increase in skills level following the collaborative radiation oncology training model used by the GO-U platform.

**Conclusion:**

Africa has the capacity to implement successful e-Learning in oncology, which is consistent with findings in previous work such as the AORTIC. Greater investment by institutions and governments is needed in terms of resources and policy changes to facilitate the implementation of effective online oncology training. Purposeful engagement of diaspora oncology health professionals with relevant cultural backgrounds as with some current collaborative efforts is highly recommended in helping turn brain drain into brain circulation.

## Background

Africa is facing an unprecedented growth in cancer burden with over 1 million new cases and 700,000 deaths per year and is inadequately prepared to meet this health challenge. The cancer burden is projected to rise dramatically over the coming years with survival rates that are amongst the lowest relative to other world regions 2030 [1]. This is exacerbated by the dearth in oncology health professionals. The limited capacity for training oncology professionals is a major contributing factor to the shortfall in human resources which stymies local control efforts within the region [2]. Consequently, there are concerted efforts to increase the African cancer workforce and promote an equitable distribution of resources [2]. Such development requires proactive efforts in each country through the provision of oncology education, training and mentorship opportunities for current and future cancer workers [3]. As Africa faces a shortage of oncology mentors, collaborative educational initiatives with mentors and trainers within and outside the continent are essential [2]. A few initiatives like the Global Health Service Partnership employ traditional face-to-face education and training options [2]. However, this often necessitates extended travel and disruption of work, causing time and financial expenses that could be prohibitive or impractical for trainers in some regions [2].

In response to these challenges, virtual oncology education and training have been suggested as tools for workforce development in Africa [2, 4, 5]. Virtual education, also known as electronic learning (e-Learning), refers to the instruction in a learning environment where the trainer and the trainee are separated by time or space, or both, and the trainer provides course content through an information and communications technology (ICT) platform that utilises course management applications, multimedia modules, the Internet, video conferencing, etc. Rather than replacing traditional training, e-Learning may be blended with traditional training, serving as a complementary mechanism to remote oncology education and instructional contents prepared by experts. However, given the obstacles to accessing Internet services in Africa including the low levels of computer literacy, inadequate infrastructure and high costs of Internet bandwidth, there are concerns that oncology trainees in African may lack the capacity and skill base for Internet-based training [2]. For example, while Internet connectivity has grown by more than 8,500% since 2000, Africa’s Internet penetration was still as low as 31.2% in 2017, below the global average of 51.7% and reflecting an inner digital divide that ranges from 4.3% in Niger to 89% in Kenya [6].

Besides connectivity issues, virtual oncology training in Africa is possible only with cultural acceptance and availability of other ICT infrastructural and managerial support systems such as electricity, e-Learning software and hardware, personnel to manage access to learning materials, technical standardisation and a framework to review learning resources [7]. In 2017, the African Organisation for Research and Training in Cancer (AORTIC) carried out a survey to assess the educational needs and e-Learning readiness of sub-Sahara African oncology staff [8]. The results highlighted deficiencies in the training of oncology health professionals in key competency areas, and supported the establishment of e-Learning to supplement ongoing traditional training to meet the deficiencies. It also showed that African oncology health professionals are technologically competent for e-Learning. However, only 15.7% of the respondents then had a bandwidth of quality to support real-time visual learning. Other analyses supported the potential for at least asynchronous learning.

Building on this work, in an effort to optimise the e-Learning experience for oncology training in Africa, educational needs assessment and e-Learning readiness studies among oncology healthcare professionals in Africa were further conducted as part of the Global Oncology University (GO-U) initiative. The investigation sought to identify and document gaps and potential challenges that might arise from implementing e-Learning. Potential solutions and a test model for effectively using ICTs for training of radiation oncology healthcare professionals in Africa are discussed. The need for ICT-powered oncology education has become even more vital in the advent of the COVID-19 pandemic [9].

## Methods

In the 2015–2016 AORTIC survey previously reported, questions assessed respondents’ educational needs and readiness for virtual oncology training. Eligible participants included all healthcare providers involved in cancer care in African countries and who were members or intending members of AORTIC. Building on the findings of this assessment, a practical test on implementing an online oncology training that is ICTs resource-intensive was conducted in 2018–2019. This included a radiation oncology training programme implemented via the ICT-powered GO-U collaborative education platform that included practical real-time treatment planning experience. A survey assessing participants’ confidence in the necessary skills needed to contour during treatment planning was administered before and after training on contouring. A further survey to assess improvements in e-Learning readiness over the years was conducted. Questions included 1) whether participants had access to computers, laptops or other technology that can allow participa tion in online lectures; 2) whether their institutions/hospitals provide free access to Internet/Wi-Fi; 3) the ability to download video files greater than 10 MB from the Internet; 4) whether they had previously taken online classes and 5) interest in online learning. The results were analysed and discussed with reference to the previous AORTIC survey.

## Results

### Assessing readiness and prospects for web-based oncology education

Readiness entails the mental or physical preparedness for some experience or action. Experts in the field have developed many models to access e-Learning readiness for institutions, trainers and learners. In assessing readiness, only trainee e-Learning readiness was assessed using the most frequently utilised components of known reference models identified in the literature. Questions focused on the five most commonly used elements across all models including (i) access to technology, (ii) technological skills/competence, (iii) capacity for self-directed learning, (iv) confidence in other prerequisite skills and (v) motivation [10].

*Access to technology* refers to the need for trainees to either have the necessary technological devices or have access to them [10]. Participants were asked to indicate if they had personal computers or any smart device meeting a minimum e-Learning requirement. Minimum computer requirements for e-Learning vary by course/content management system or learning management system that is used to deliver the course materials. However, the minimum technology required for this study is the minimum necessary for Skype communication, which is a computer or smart devices with at least a 1 GHz processor and 256 MB of RAM installed. Respondents were also asked to indicate if they had or could afford Internet connectivity for an online course. A 56 Kbps connection speed was deemed feasible for asynchronous virtual oncology education and a minimum of 1 Mbps broadband connection speed for synchronous visual/audio learning.

*Technological skills/competence* signifies the trainee’s self-efficacy in using the computer, the Internet and other smart devices [10]. Survey respondents were asked to indicate if they were confident in using computers or smart technologies, and if they could efficiently utilise the Internet and software for virtual learning. Each further stated that they would be comfortable using a computer or device several times a week to participate in an online course.

*Capacity for self-directed learning* entails a process in which individuals take the initiative, with or without the help of others, in diagnosing their learning needs, formulating learning goals, identifying human and material resources for learning, choosing and implementing appropriate learning strategies and evaluating learning outcomes. Respondents were asked to indicate if they have all the computer literacy skills needed to find learning sources, determine which strategies they should employ, evaluate themselves and navigate online learning platforms and processes for themselves. Respondents were asked to indicate the availability of trained teaching and ICT personnel in their institutions who could assist them should they be unable to self-direct.

*Motivation* is the students’ willingness and eagerness concerning attending classes via online or electronic methods [10]. Motivation was gauged by comparing their preference between e-Learning modalities and face-to-face learning. Respondents were further probed to indicate if they ever took an online course. Estimations for motivation were based on the percentage of respondents who had completed the courses.

*Confidence in prerequisite skills and yourself* is a combined component, which comprises one’s own trust towards the skills required to be successful in e-Learning and towards oneself [10]. Respondents were asked to indicate if they felt that they were confident in their capacity to communicate effectively and at pace with course participants and instructors (in both writing and speaking) via an online platform.

**Figure 1.**
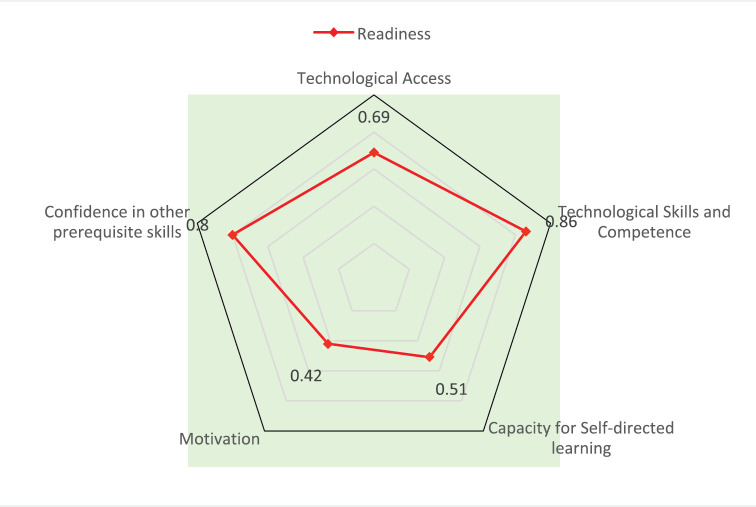
Trainee’s readiness for virtual oncology education in Africa.

In the previously reported AORTIC survey conducted in 2015–2016 [8], 110 out of 128 (about 86%) respondents had personal computers of the minimum specification, 86 had computers in their workplace and 95 had other technological devices such as tablets and phones that could adequately support e-Learning. All respondents had Internet connectivity, but only 20 (15.7%) had a quality connection greater than 1 Mbps of bandwidth speed to support synchronous e-Learning. Exactly 113 out of 127 respondents had the necessary skills to operate the computer or smart device for e-Learning. The same number felt capable of navigating the Internet for learning activities such as research, assignments and accessing online libraries, while 101 respondents felt comfortable using the Internet several times a week for an online course. Although 113 thought they had necessary computer skills, only 47 out of 128 respondents felt they had all the computer literacy skills needed to function as an independent learner. However, 48 indicated they could easily access technical support from their institutions when required and 56 reported they could seek administrative help from e-Learning experts. This makes synchro nous self-directed learning feasible for 48 and asynchronous self-directed learning possible for 107. Only 43 (33.5%) oncology staff preferred e-Learning over the traditional face-to-face training modality. Although 25 participants worked or were trained in schools supportive of online courses, only 11 participants indicated ever taking an online-based course. All 11 candidates completed their courses. All courses blended face-to-face and e-Learning and none (0) were strictly online oncology courses. One hundred and one participants indicated confidence in their ability to communicate effectively with course participants and instructors (in both writing and speaking) via an online platform. Based on these responses, the weighted averages for technological access, technological skill and competence, capacity for self-directed learning, motivation and confidence in other prerequisite skills were 69%, 86%, 51% 42%, and 80%, respectively (see Figure 1).

**Figure 2.**
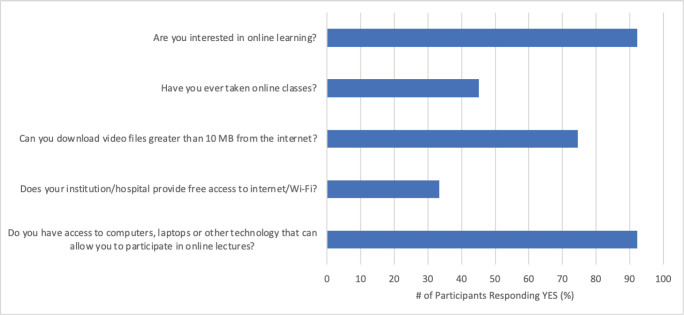
ICT assessment for oncology health professionals in sub-Saharan Africa (*n* = 51).

Relevant results from the following (2019–2020) survey conducted as part of the GO-U initiative are shown in Figure 2. The results show that over 92% of the respondents had access to computers, laptops or other technology that can allow them to participate in online courses. This was higher than the results of the AORTIC survey, but is consistent with the overall assessment that an overwhelming percentage has the hardware to participate in online learning. While nearly 100% of the respondents had access to the Internet, also consistent with the AORTIC survey results, only about 34% of the respondents indicated that their institutions provided free access to the Internet. Meanwhile, 75% indicated that their Internet bandwidth was sufficient for downloading large-size files over 10 MB. About 92% of the respondents indicated they were interested in online learning, with 45% indicating they had already participated in some form of online learning course. An obvious limitation of this survey and the AORTIC survey is that this comes from a broad representation of respondents from different institutions and the actual situation may vary from institution to institution, depending on the location and resources.

Taken together with the results of the previous AORTIC survey, the findings indicate capacity for African health professionals to participate in online learning courses.

There is need, however, for greater investments by institutions in providing resources to support online education that could benefit oncology training. With the COVID-19 pandemic, there is indication that there is even more interest in online oncology education. Prior to the pandemic, many institutions in Africa, as many in the earlier survey, were less interested in online education. Our recent survey indicates a significant jump in interest and many African institution partners are not asking to be part of the collaborative GO-U online training platform.

## Experience on the use of ICTs via the GO-U training platform

**Figure 3.**
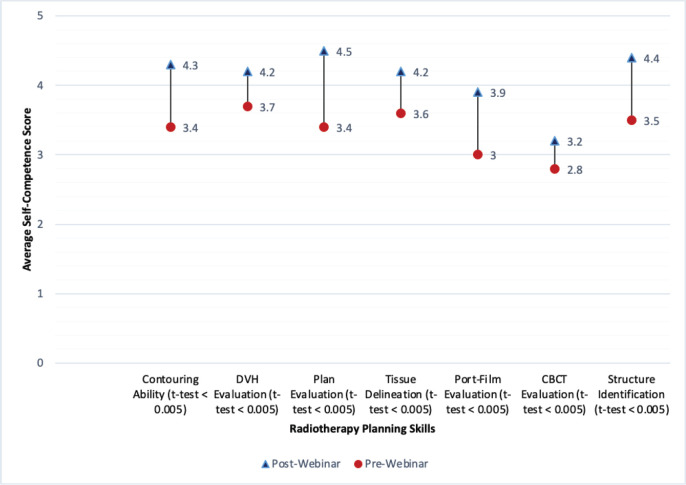
Difference in competence scores pre- and post-online training session on radiotherapy contouring. A score of 5 signifies full competence in prerequisite contouring skill and ‘1’ is complete incompetence. Study participants rated themselves using pre- and post-class questionnaires.

Radiotherapy is employed in the treatment of over 50% of cancer patients. Following the assessment of readiness, a radiation oncology training programme was implemented via the ICTsGO-U training platform. A survey assessing participants’ confidence in the necessary skills needed to contour, which is crucial in radiation oncology treatment planning, was administered before and after training on contouring. Self-confidence on each skill was self-reported on a scale of ‘1’–‘5’. A general average score for each component was calculated as a summation of scores divided by the total number of respondents. There was a marked improvement in the self-confidence to contour after a single 1-hour webinar session (Figure 3). While this is simply an indication of potential, the results of this assessment suggests the value that ICT-enabled education and training can bring about the transfer of knowledge, skills, abilities and even perception in oncology education and training. In the case of the GO-U contour training example, participants’ perception of their own abilities was positively affected after a 1-hour webinar, which could lead to a greater openness to additional training opportunities, combating some of the hesitation towards e-Learning identified in other assessments.

## Discussion and recommendations

Africa is virtually ready for ICT-powered oncology education. Internet access among the oncology staff is 100%, significantly higher than the 31.2% of sub-Saharan Africa [6]. This high access is likely because oncology professionals are among the most educated in their societies who are also among the top income earners. This gives this population a greater likelihood of benefitting from ICT-related opportunities [11]. The high occurrence of personal computer or technological device ownership negates the need for e-Learning centres for cancer training. Low quality of the Internet connection, nevertheless, could profoundly affect the standard of training and modality of virtual learning a platform can adopt. Real-time, video-based synchronous learning, which involves more intense interaction between trainees and trainers, may not be feasible for some where the bandwidth is low. As an observation, the low bandwidth impeded the display of real-time video lectures for some participants of the global radiation oncology course. The solution may be to adopt an asynchronous e-Learning system where videos’ contents are recorded and made available for downloads. Asynchronous e-Learning may also have the advantage of being able to include procedural training processes in oncology such as contouring or surgery that may not be possible in real-time lectures. Greater investments by institutions and governments to provide access to the Internet with sufficient bandwidth are recommended, along with partnerships with major telecommunication companies like MTN. It is worth noting that, while synchronous e-Learning could improve trainee’s engagement and motivation, it decreases the ability to process information [11]. Asynchronous learning, on the other hand, is more useful for cognitive participation which is more reflective since downloaded contents can replay over and over again [11]. Both approaches are recommended in content delivery.

Lack of technical skills is often regarded as the most significant barrier to participation in e-Learning since learners often experience fear and anxiety when using computers and programmes with which they have no familiarity. Adequate user training is usually required prior to implementation of a virtual learning programme and is often the most critical success factor in any e-Learning programme [12]. Based on the results and experience, oncology staff may not require further technical training except for virtual training requiring advanced software. The challenge, however, lies in motivation and capacity for self-directed e-Learning. There are indications that this has improved significantly during the COVID-19 pandemic as seen by many adopting this [9]. Additional software and hardware training with technical support teams within the e-Learning interphase may be useful for advanced virtual instruction. Some course contents can be self-paced for trainees who are unable to attend as scheduled.

The 21st century presents unprecedented opportunities for access to web-based learning. Mobile connectivity stands at 7.6 billion globally, and mobile broadband penetration has significantly increased in the past decade. Smartphone penetration is already at 48%, and predictions anticipated 5.6 billion smartphones by 2020, with 90% of the users in LMICs. These predictions were reflected in findings from our recent survey involving diverse participants, which showed that 92% of the responders had access to ICT devices and about 75% could download more than 10 MB from their Internet. There are still limitations in the sense that access to the type of high bandwidths needed for live sessions may not be available to the masses, but many mobile uses and those on other devices including computers and tablets, are able to support asynchronous learning options that often require lower bandwidths. This highlights the importance of working with collaborators including institutions and organisations that can extend strong web access options to participants in LMICs as needed for live lectures and underscores the importance of diversified delivery options. In this way, live instruction can provide interaction with presenters offering direct access to experts who are experienced in the material and methods they are teaching. In addition, recorded lectures, lecture slides and other material that can be accessed on lower bandwidths can be used for the purpose of instruction with support from local partners, as well as stored as part of a library of resources to be consulted for review and clarification.

The challenges posed by the exponentially growing cancer burden in Africa are both daunting and urgent. Here is where the use of ICTs can offer opportunities to make strides in education and training for health professionals in low-resource countries. Studies have shown that key elements are in place for utilising ICTs to enable high-impact collaborations in response to the growing global cancer burden. This can be particularly useful in radiation oncology where partners from different nations with diverse backgrounds can cooperate to bridge the chasm that currently exists when it comes to the availability of safe, effective radiation therapies as an accessible cancer treatment option for LMICs [13].

Through collaborations with diverse stakeholders, facilitated by ICTs, life-saving and life-enhancing health solutions are already being implemented around the world. For clinicians, researchers, educators and advocates who give their time and talents to make these services possible, such as through GO-U, ICTs provide an opportunity to change the world without traversing the world. Former barriers posed by time and distances are no longer an excuse (albeit legitimate) for the inability to contribute to global health gains. Many seasoned scholars and practitioners understand the value of knowledge and skill sharing as a responsibility and gesture of goodwill to extend and enhance practice in the professions they hold dear. ICTs provide an avenue for such professionals to have meaningful impact worldwide, while leaving a philanthropic legacy. Finally, global populations are afforded the opportunity to make profitable investments, focus on global policies that promote national priorities and encourage international and intercultural exchanges that ultimately foster goodwill, setting the stage for global gains that extend beyond global health gains. There are opportunities for win-win scenarios where all stakeholders both contribute and benefit from collaborative global education and training endeavours powered by ICTs.

Some institutions are moving towards greater adoption of ICTs in education and training. One university that has been an excellent example of online education across different African countries is the ICT University. The university has its presence in Cameroon, Nigeria and Uganda and is rapidly expanding across Africa, while collaborating with academic institutions in Europe and the USA to advance the training of health professionals in Africa, including oncology [8, 14, 15]. The GO-U has also a growing partnership with faculty across different institutions in Africa and leading world institutions in the USA and Europe focused on oncology education and training, with certificate courses offered online and courses for long-term degree and residency programmes conducted in partnership with local institutions in different African countries. This model is expected to accelerate in growth and impact during the COVID-19 pandemic and beyond. The cost of these programmes is expected to vary from free to the similar costs paid in different countries. In one example with the University of Nairobi [9], this is expected to lead to higher quality education with oncology experts and collaborating with faculty from world-leading institutions in the USA and Europe contributing to fill gaps.

Box 1. **Summary of the recommendations for advancing online oncology education for Africa.**

Academic and healthcare institutions should invest more in infrastructure and resources to support online education in oncology and review their curricula to accommodate for complementary online training and continuous education to fill knowledge gaps.

Governments should support institutions and healthcare organisations in their countries through policies, additional resources and subsidies to advance online education.

Greater engagement of diaspora oncology professionals is highly recommended, allowing them to teach online, e.g., through adjunct faculty appointments to support the local institutions, in an approach that could help turn brain drain into brain circulation.

Greater collaborations are recommended among African institutions and with leading institutions outside of Africa as highlighted by the GO-U model.

Win-win partnerships with telecommunication companies that can provide additional support for online education is recommended.

Professional societies like ASCO with many education resources should increase collaboration and support for African professional societies like AORTIC to increase access to online oncology education.

Collaborations with organisations and charities like *e*cancer and non-profit organisations like e-Oncologia are recommended.

Global Oncology Education powered by ICTs should be synergised where possible with other tele-oncology and research initiatives leveraging ICTs [16].

It is worth noting that when health crises like the COVID-19 pandemic affect populations that possess the resources and access to quality care, innovative technologies, advanced equipment and highly trained professionals, such crises become an opportunity to activate these available resources to serve the affected communities. In the case of communicable diseases, these resources may be deployed to neighbouring communities as part of a preventive or comprehensive approach to stemming the impact of the communicable disease [9]. However, when non-communicable diseases affect populations with limited access to care, technologies, equipment and professionals, goodwill is often not enough to surmount the real challenges posed by limitations of time, distances and resources available for external aid from high income countries (HIC) to low- and middle-income countries (LMICs). This is often the case with cancer. Many LMIC patients and their families suffer not only because they lack the resources to afford quality care, but also because the resources needed to provide care are not readily available within their communities. Moreover, access to resources in HIC is extremely limited by cost, time, distance and travel-related barriers. The GO-U programme and other initiatives with *e*cancer and professional societies like the American Society of Clinical Oncology (ASCO) are examples of growing opportunities that could significantly advance global health. So while needs abound and resources are limited, cancer education and training in Africa now has unprecedented opportunities for human resource development in cancer education and training by leveraging ICTs and mutually beneficial collaborations. This could include participation of diaspora oncology health professionals, turning brain drain into global health gain. Box 1 includes a summary of the recommendations for advancing ICT-powered oncology education for Africa.

## Conclusion

Africa is ready for online oncology education. Collaborative approaches that offer online learning in partnership with local Institutions in Africa provide a viable model. Strategic engagement with the diaspora provides a major opportunity to turn brain drain into brain circulation. Professional societies like AORTIC also have a crucial role in advancing online education, working in partnerships with other leading professional societies like ASCO and the European Society of Clinical Oncology.

## Funding

This work was partly supported by the Brigham and Women’s Hospital Biomedical Research Institute.

## Conflicts of interest statement

There are no conflicts of interest.
